# Correction: An Unexpected Sequence of Events: Mismatch Detection in the Human Hippocampus

**DOI:** 10.1371/journal.pbio.3000442

**Published:** 2019-08-12

**Authors:** Dharshan Kumaran, Eleanor A. Maguire

## Notice of republication

This article was republished on July 30, 2019 to correct a figure which contained images that were not available for publication under the Creative Commons Attribution License. Fig 1 has been recreated by the authors using open source images. The figure legend for Fig 1 has been updated to reflect this change.

References to this figure in the Materials and Methods now state that Fig 1 is visually similar to the images used in the experiments, but not identical.

Please download this article again to view the correct version of the figure.
